# *Mycobacterium smegmatis* Expands Across Surfaces by Hydraulic Sliding

**DOI:** 10.1111/1758-2229.70214

**Published:** 2025-10-28

**Authors:** Eric J. G. Pollitt, Oliver Carnell, Egbert Hoiczyk, Jeffrey Green

**Affiliations:** ^1^ School of Biosciences University of Sheffield, Firth Court, Western Bank Sheffield UK

**Keywords:** biofilm, colony morphology, dendrites, mycobacteria, pellicle, pseudofilaments, sliding motility

## Abstract

Passive sliding motility allows *Mycobacterium smegmatis* to spread over soft agar surfaces. Usually, bacterial growth and reduced surface adhesion push individual bacteria outwards, resulting in circular colonies; however, more complex, dendritic colonies have also been reported. Although we could readily reproduce the circular morphotype, our non‐circular colonies (hereafter digitate colonies) differed from dendritic colonies. Digitate colonies were characterised by centimetre‐long, linear protrusions consisting of surface pellicle and inner biofilm components surrounding a central channel that was filled with a free‐flowing suspension of *M. smegmatis* and aggregates. Time‐lapse microscopy showed that the expansion of the fluid‐filled channel resulted in lengthwise extension of the protrusions without any perceptible bacterial growth. These observations reveal a novel type of sliding motility (named hydraulic sliding) associated with a distinct colony structure and the apparent generation of force by expansion of a liquid core. The presence of a pellicle (a floating biofilm) generated without an initial liquid‐air interface suggests that a previously unknown mycobacterial behaviour that could be important for colonisation and virulence has been discovered.

## Graphical abstract

We present a new form of passive motility in mycobacteria, named hydraulic sliding, in which liquid expansion is the cause of motility. This study also reveals new ways mycobacteria can form biofilms and colonise 3D substrates, aspects of mycobacterial biology that are important for understanding infection, pathogenesis and vaccine development.

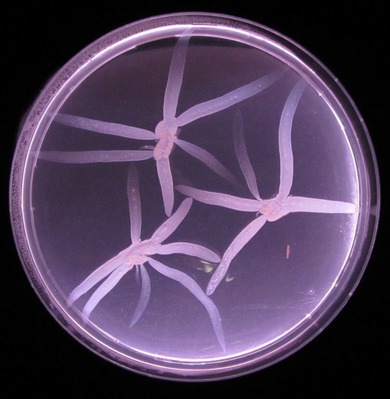

**Abbreviations**
GPL: glycopeptidolipids
HEX: hexamethyldisilazane
IR: infrared spectroscopy
SDS‐PAGE: SDS‐polyacrylamide gel electrophoresis
SEM: scanning electron microscopy

## 1 Introduction

*M. smegmatis* is a model mycobacterium, often studied to gain insights into the biology of major pathogens such as *Mycobacterium tuberculosis* . *M. smegmatis*, like other mycobacteria, is non‐flagellated, but it can move across some surfaces by a process called sliding (Martínez et al. 1999). Sliding is a form of passive motility that was originally defined as ‘surface translocation produced by expansive forces in a growing culture in combination with special surface properties’ (Henrichsen 1972). Thus, sliding motility often results in circular colonies, as growth combined with secreted factors to lower surface adhesion results in bacterial cells being pushed outwards without any directional bias (Henrichsen 1972; Hölscher and Kovács 2017). Martínez et al. (1999) reported that *M. smegmatis* strain mc^2^155 grown on 7H9 medium solidified with agarose produced circular colonies with edges consisting of a monolayer of cells that moved outwards as a compact group. Within these circular colonies, the bacteria formed structures called pseudofilaments consisting of small branching groups of cells held together by an amorphous material. No active motility was observed and hence this behaviour was classified as sliding motility. All subsequent studies of the genetic basis of *M. smegmatis* sliding have used this agarose‐based motility assay (Recht et al. 2000; Recht and Kolter 2001; Gopalaswamy et al. 2008; Jamet et al. 2015). Under these conditions, sliding motility is primarily dependent on the presence of glycopeptidolipids (GPLs), a component of the cell envelope that is implicated in virulence and drug susceptibility in pathogenic mycobacteria (Martínez et al. 1999; Recht et al. 2000; Schorey and Sweet 2008; Mukherjee and Chatterji 2012; Chakraborty and Kumar 2019).

Remarkably, Martínez et al. (1999) also reported that when Difco agar was used to solidify 7H9 medium, *M. smegmatis* did not form circular colonies, but rather formed colonies with long, thin dendrites. This new morphotype was thought to arise as a result of sliding motility because the leading edges of the dendrites consisted of a monolayer of cells that moved outwards as a unit, with no evidence of active motility. Non‐circular morphologies like dendritic colonies arising from passive sliding are uncommon. When we investigated the dendrite colony morphotype of *M. smegmatis* , we discovered a new colony form (digitate colonies), which exhibited centimetre‐long protrusions containing a free‐flowing suspension of bacteria covered by a pellicle. It appears that fluid accumulation in the channel enabled expansion of the finger‐like protrusions and hence a new form of sliding motility, hydraulic sliding, is proposed.

## 2 Results and Discussion

### 2.1 Observation of a New *Mycobacterium smegmatis* Colony Morphology

**FIGURE 1:**
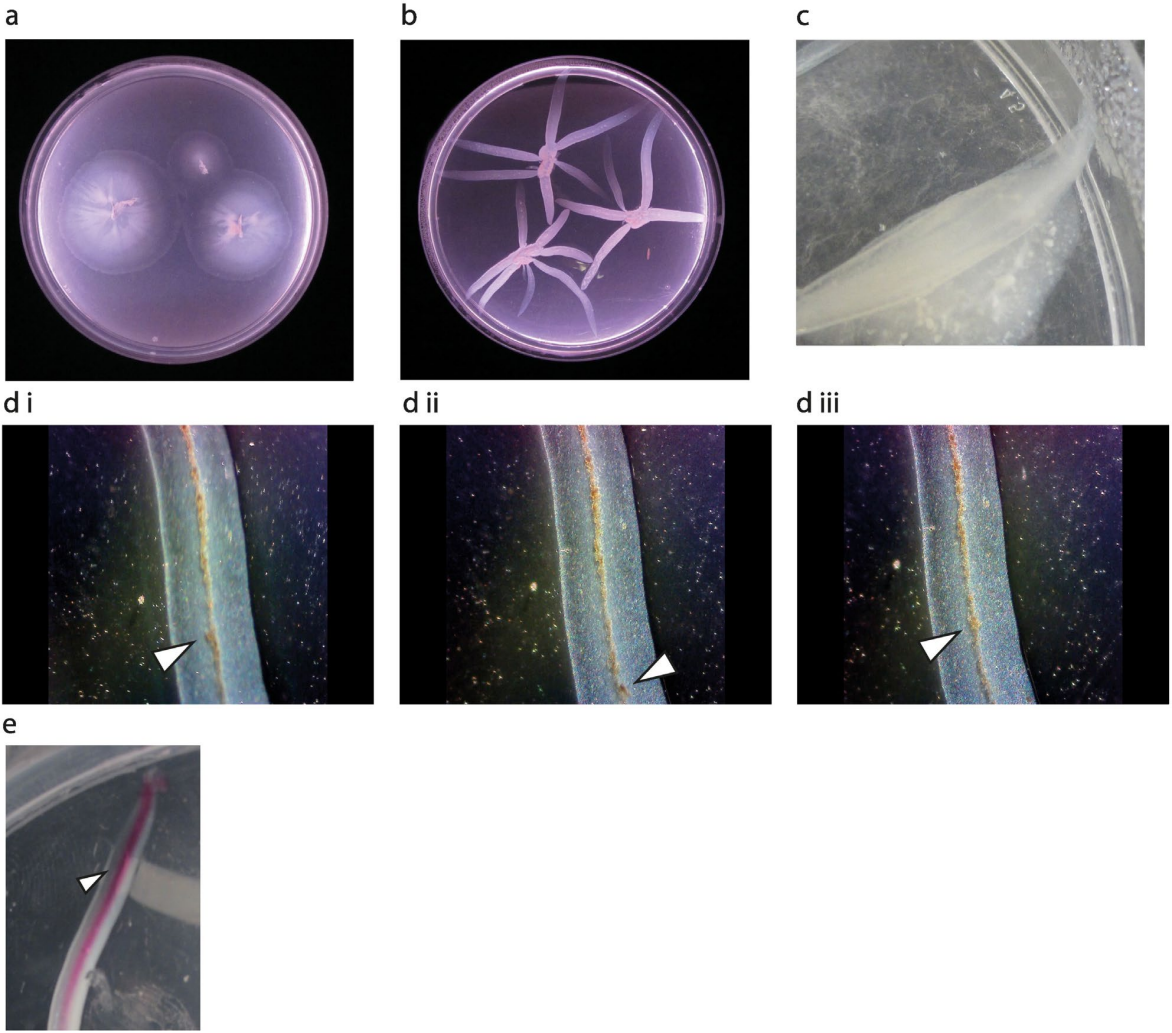
Observation of a new *M. smegmatis* colony morphotype. (a) Circular colonies on 0.4% agarose medium 3 days post‐inoculation. (b) Digitate colonies on 0.2% Noble agar medium 3 days post‐inoculation. (c) A digitate colony protrusion extending directly up the meniscus of the agar plate. (d) Selected sequential images (i–iii) from Video S1 of the fluid filled central channel located within the digitate colony protrusions observed under a dissecting microscope (×4 magnification). As the plate was rocked the aggregates moved within the central core, the large aggregate indicated by the arrows was tracked up and down the central channel (see arrows and Video S1). Nothing outside the central channel moved. (e) Injection of phenol red into the central channel of a typical digitate colony. Injecting 5 μL of phenol red (arrow indicates site of injection) followed by tilting the plate allowed the dye to move through the liquid‐filled channel. The plates were prepared as described in the Supporting Information: Experimental Procedures. Briefly, all media were autoclaved and cooled to ~55°C and then dispensed (25 mL aliquots) into 9 cm Petri dishes. The plates were left covered in a laminar flow cabinet overnight (not running) to set and were then inoculated with colonies taken directly from an *M. smegmatis* stock plate. The plates were sealed with Parafilm and incubated at 37°C in a humidity‐controlled incubator. The circular colony medium was agarose (Sigma, A5093; 0.4%) and 7H9 (Oxoid, 1.175 g) in 225 mL of distilled water. The digitate colony medium was prepared using Noble agar (Oxoid; 0.2%) and 7H9 (Oxoid, 1.175 g) in 225 mL of distilled water.

In initial experiments we readily reproduced the *M. smegmatis* circular colony form on agarose medium, as reported by Martínez et al. (1999) (Figure 1a). The edges and cellular structures of these circular colonies were similar to those reported by Martínez et al. (1999), even though there were some differences in structure at the centre of our circular colonies. This type of variation has been observed by others, and we also note that *M. smegmatis* biofilms develop ultrastructures over time (wrinkles, etc.) and this might account for some of these differences (Ojha et al. 2005; Báez‐Ramírez et al. 2021). Thus, whilst we were able to generate circular colonies similar to those of Martínez et al. (1999), we failed to replicate the dendrite morphotype using Bacto agar. Very occasionally short branching dendrites were observed, but the long dendrites previously reported were not formed, leading us to test the effects of other medium solidifying agents.

Colonies of *M. smegmatis* with centimetre‐long protrusions radiating from their centres were observed when Noble agar was used as the solidifying agent (Figure 1b). Noble agar is known to be much purer than Bacto agar (containing less ash and inorganic impurities) and confers higher gel strength (Zimbro et al. 2009). The observed colony protrusions were much broader and straighter than those of the dendritic colonies previously reported (Martínez et al. 1999). Protrusion formation appeared to be inhibited by higher agar concentrations (Figure S1). These observations served to illustrate the importance of surface composition as a determinant of bacterial behaviour. To distinguish the colonies formed on Noble agar from the previously observed dendritic colonies (Martínez et al. 1999), we refer to this new morphotype as digitate colonies.

### 2.2 Digitate Colonies of *Mycobacterium smegmatis* Have Liquid Filled Protrusions

The digitate colonies had some fundamentally different characteristics from any previously reported *M. smegmatis* dendritic colonies (Martínez et al. 1999). Thus, Martínez et al. (1999) reported that after 3–4 days, long thin finger‐like extensions from the colony were observed, with occasional branching. Furthermore, the tips of the extensions were preceded by a monolayer of cells with no individual movement. By contrast, the digitate colonies reported here developed over 3 days from inoculation of the plate, with on average, 3–5 broad protrusions emanating from the centre of each digitate colony (Figures 1b,c and S2). These protrusions differed from the dendritic colony morphology in that: (1) each protrusion was linear with no branching; (2) the protrusions were broader; and (3) within each protrusion there was a core of mobile liquid that contained suspended bacteria and larger aggregates (Figure 1d). The liquid core was fully mobile within the protrusions and extended almost to their tips. The liquid was retained when the plate was briefly inverted or rocked (Video S1). Introduction of Phenol Red to the liquid core of a protrusion followed by rocking the plate showed that the dye flowed rapidly through the liquid core of the protrusions of the colony and was retained within them (Figure 1e). Fluid‐filled colony protrusions have not been reported previously for any *Mycobacteria* or Gram‐positive bacteria; however, some strains of *Roseobacters* (Gram‐negative) have been reported to form liquid‐filled channels (Bartling et al. 2018).

Application of IR and mass spectroscopies to samples of channel fluid showed that it differed from samples of liquid cultures (Figures S3–S5; Table S1). Some compounds putatively identified could hypothetically be involved in the formation of digitate colonies, such as p‐HBAD (*p*‐Hydroxybenzoic acid derivatives; associated with phenolic glycolipids, a major component of the mycobacterial cell envelope). SDS‐PAGE analysis did not reveal any major differences in protein content between *M. smegmatis* liquid culture and the digitate colony extracts (though there may be differences in the proportional expression of certain polypeptides) (Figure S6). Nevertheless, it has been shown that the digitate colony channel fluid can be readily extracted and characterised, which should enable further studies to identify the major components of the channel fluid. If there are novel products in the channel fluid, this could be interesting because compounds required for movement are often important virulence factors or act as antibiotics (Kearns 2010; Otto 2014; Hölscher and Kovács 2017).

### 2.3 Cellular Organisation

**FIGURE 2:**
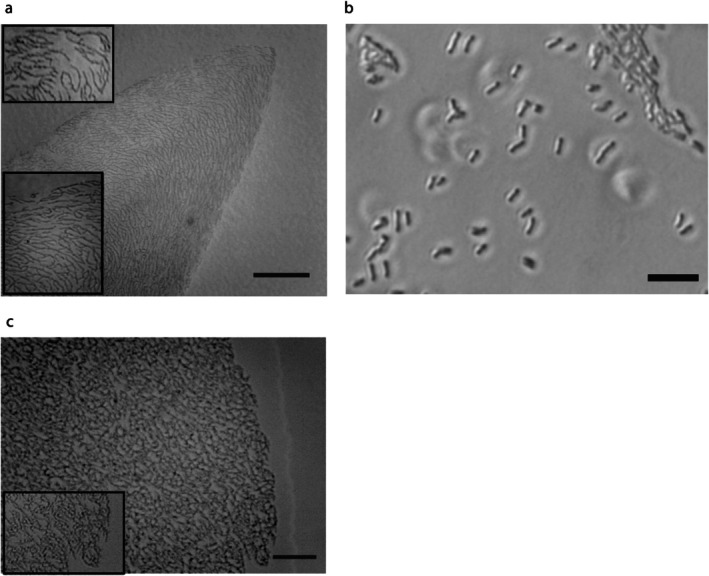
Light microscopy images of *M. smegmatis* sliding colonies. (a) The tip of a digitate colony observed using a phase contrast microscope. The bacteria are arranged as branching pseudofilaments that broadly align with the curve of the protrusion tip. Scale bar: 100 μm. A magnified subsection of the pseudofilaments is also shown; the top image is the tip and the bottom image is the side of the colony (edge to top of image) showing how the pseudofilaments align with the edge of the colony. (b) Image of *M. smegmatis* cells grown in 7H9 liquid culture on a glass slide showing that the cells do not branch. Scale bar: 20 μm. (c) The edge of a circular *M. smegmatis* colony showing the pseudofilaments are unaligned. A magnified subsection of the pseudofilaments is shown bottom left. Scale bar: 50 μm. The plates used were as described in Figure 1 with 0.25% noble agar in (a), 7H9 liquid medium in (b), and 0.4% agarose in (c).

Examination of the tips and edges of the digitate colonies by light microscopy revealed the presence of a monolayer of mycobacterial pseudofilaments arranged as branching chains of less than 20 individual cells (Figures 2a and S7). The circular colonies also had a monolayer of branching pseudofilaments. Branching pseudofilaments (groups of cells attached by amorphous material but with separate cell walls) were previously observed in circular colonies (Martínez et al. 1999), and those observed in the digitate colonies are visually similar; note that these pseudofilaments do not occur in liquid culture (Figure 2b). In the digitate colonies, the bacteria broadly aligned with the edge of the protrusion and curved around at the tip (Figure 2a). The pseudofilaments of circular colonies were not aligned and distributed randomly, as seen previously by Martínez et al. (1999) (Figure 2c). We also note that, as reported by Martínez et al. (1999), the circular colonies have a layer of slime/fluid that surrounds and precedes the colony (Figure 2c). However, the digitate colonies do not have the same extensive fluid release.

The branching *M. smegmatis* pseudofilaments resemble the branching hyphae/vegetative mycelium that form to penetrate growth substrates in the closely related *Streptomyces* (Scherr and Nguyen 2009) and might suggest changes in the process of cell division (Flärdh and Buttner 2009). Furthermore, a branching pattern is generally poor for moving over surfaces, raising the question as to why this morphology is adopted during mycobacterial sliding motility (Young 2006).

### 2.4 Biofilm Components of Digitate Colony Protrusions

**FIGURE 3:**
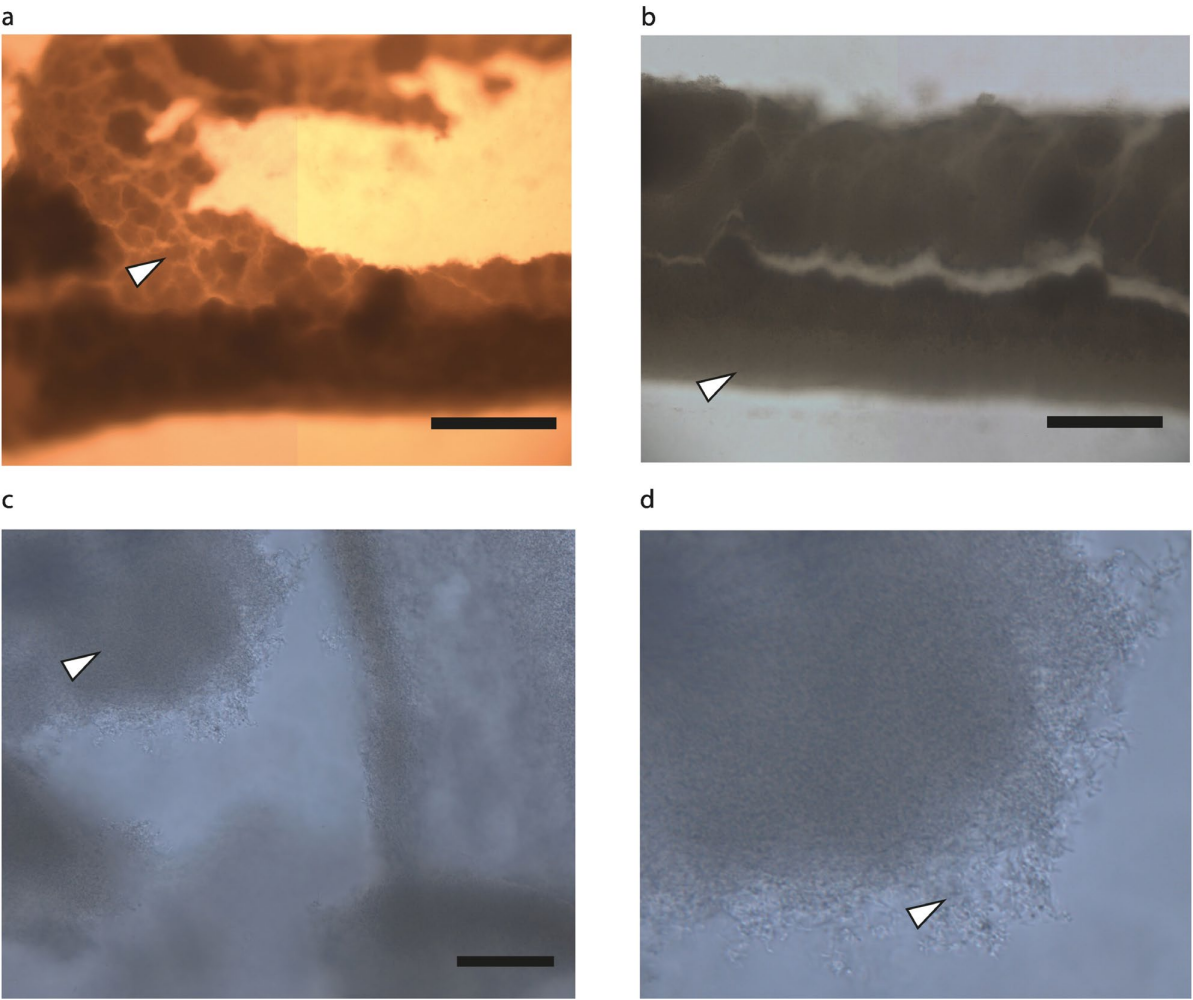
Images of the pellicle enclosing the protrusions of digitate colonies. (a) Representative section of pellicle is indicated by the arrow; at the bottom of the image, the pellicle has folded over. Scale bar: 500 μm. (b) Another section of the pellicle showing a protrusion edge. The edge has a different consistency from the rest of the pellicle (arrow). Scale bar: 500 μm. (c) Pellicle fragment showing the edge consisting of disorganised tangled bacteria. The pellicle itself appears relatively thick (representative section indicated by arrow). Scale bar: 100 μm. (d) Enlarged section of (c) showing that it is many layers thick, and where it is exposed, the bacteria formed disorganised but dense structures.

Digitate colonies were flattened and observed under a light microscope to investigate the nature of the upper surface of the protrusions, which proved to be a comparatively thick pellicle, resembling the mycobacterial pellicles generated on the surface of liquid cultures (Figure 3; Pang et al. 2012; Parvez et al. 2018). By visual inspection, we estimated this layer to be formed from at least 20 cell layers; certainly, more than one layer, but much less than that seen in the standard *M. smegmatis* pellicle assay. The pellicle had a different hue from the edges of the colony, and the edges appeared to be more strongly attached to the agar, tearing away from the main pellicle when the structure was disrupted. We did not observe any of the wrinkling associated with mature *M. smegmatis* pellicles on the surfaces of liquid cultures (Ojha et al. 2005). Some mycobacteria develop as dense connected cords that are associated with virulence (Kalsum et al. 2017), and although *M. smegmatis* does not possess the cording factor (Julián et al. 2010), it is possible that the dense biofilms in the digitate colonies could form as a result of some related cording behaviour or specialised clumping.

When the pellicle was removed, a fixed layer of bacteria was seen below the mobile liquid suspension (Video S2). Unfortunately, the channel liquid prevented detailed analysis of this lower biofilm layer; flattening the sample left no observable structures, and hence this layer appears less robust than the upper pellicle. Although GPLs, which are required for sliding motility, are not covalently attached to mycobacteria, they are considered to be retained at the cell surface and so are unlikely to be present in the channel fluid, but nevertheless could contribute to the formation of the surrounding biofilm (Recht et al. 2000). It is noted that mycobacteria produce slime of unknown composition when engaged in sliding, but this tends to be found all over the colony surface and not within the colony itself (Martínez et al. 1999; Arora et al. 2008); we did not observe extensive slime release in the digitate colonies, as has been seen in circular colonies (see above).

### 2.5 New Protrusions Emerge When the Colony Tips Are Physically Manipulated

**FIGURE 4:**
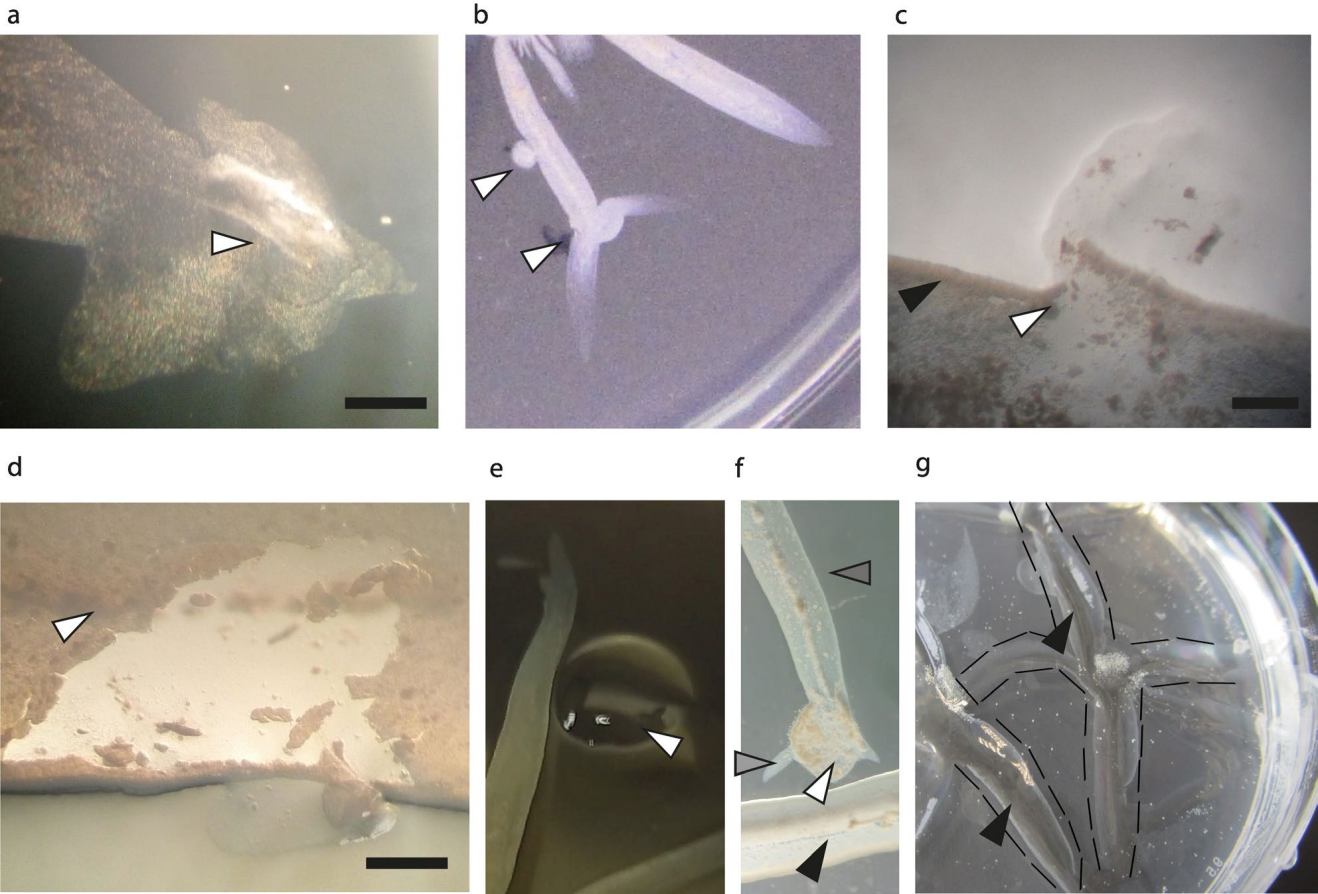
Disruption of a digitate colony. (a) Disrupting the tip of a protrusion resulted in fluid release (arrow indicates the centre of disruption site). Scale bar: 1 mm. (b) Incubation after disrupting the tip of a protrusion resulted in the formation of new protrusions, as if starting a new colony (bottom arrow). In contrast, when the middle of the protrusion was damaged a bleb formed (top arrow). (c) Disruption of the side of a protrusion showed that there is a layer of cells retaining the fluid (white arrowhead marks the site of nicking the edge, note the specialised biofilm edge of the protrusion can be seen here [black arrowhead]). Scale bar: 1 mm. (d) The pellicle on top of the protrusion can be removed, leaving intact both the central core and the edge where it comes in contact with the agar. The pellicle can be seen here due to the light angle used in the dissecting microscope (arrow). The fluid still flowed through the central core. Scale bar: 1 mm. (e) A droplet (20 μL) of channel fluid (white arrowhead) placed next to a protrusion resulted in a slight deviation in the direction of extension, but did not cause any gross morphological changes. (f) A droplet of digitate colony suspension (white arrowhead) placed close to a mature protrusion (black arrowhead) resulted in formation of a new colony with small protrusions extending towards (dark arrowhead) and 1 or 2 large protrusions (light grey arrowhead) extending away from the existing protrusion. (g) When a colony was extracted it left a V‐shaped groove in the agar that penetrated to nearly the base of the Petri dish (white lines: Edge of the digitate colony, black arrow: Bottom of the groove in which the protrusion sits). Digitate colony medium and incubation conditions were as described for Figure 1. For further details of the microscope settings, please see Supporting Information: Experimental Procedures.

Damaging the tips of the protrusions resulted in the emergence of fluid, which was at least partially immiscible with the surface of the agar plates (Figure 4a); water droplets spotted on agar dissipated more rapidly than the channel liquid, suggesting that the latter is compositionally distinct from water due to the presence of cell‐secreted materials. Damaged tips acted as origins for multiple protrusions (Figure 4b). Disruption of the edge of a protrusion resulted in a bleb, and if heavily damaged, a new protrusion began to develop from the lesion (Figure 4b, break shown in Figure 4c). Scratching through the pellicle completely also resulted in new protrusions that extended from the site of damage whilst the original protrusion continued to extend. The pellicle on top of the protrusions could be removed whilst retaining the fluid within the protrusion channel (Figure 4d, Video S2). The pellicle grew back 24 h after removal with no obvious scar. Repeated removal of the pellicle did not prevent the protrusion from extending.

We also tested the response of the colonies to added *M. smegmatis* and filtered protrusion channel fluid, as motile bacteria avoiding other colonies is an important behaviour. The *M. smegmatis* protrusions generally stopped immediately before hitting other protrusions (Figure 1b), although occasionally fusion was observed. Spotting filtered protrusion channel fluid as close as possible to a protrusion without disrupting the biofilm edges resulted in the protrusions bending inwards around the droplet (Figure 4e). However, there were no further observable effects, and once the droplet had dissipated the protrusion edge straightened. When a droplet of channel fluid was placed close to the tip of a protrusion, new protrusions were formed upon contact. These new protrusions radiated away from the site of contact, reminiscent of the formation of a new digitate colony. However, when the droplet was spotted more than 1 cm in front of a protrusion it was not recognized and the protrusion passed straight through it as it extended across the surface. When a suspension of *M. smegmatis* taken from the channel of a protrusion was spotted next to another protrusion, a new colony was formed, which did not merge with the old colony (Figure 4f); at best, abortive protrusions were formed in the direction of the old colony, whilst 1 or 2 protrusions extended away from the old colony.

### 2.6 Digitate Colonies Form V‐Shaped Clefts in the Agar Surface

Removal of digitate colonies from the agar surface revealed the presence of V‐shaped clefts beneath the protrusions that extended almost to the bottom of the agar plate (Figure 4g). SEM imaging of the internal face of the V‐shaped clefts showed that the agar had been modified compared with the surrounding agar (Figure S8). When agar is desiccated, it becomes fibrous (as seen at the top of Figure S8a), but this was not observed inside the cleft, where it appeared to be composed of more aggregated strands that penetrated into the surrounding agar (Figure S8b,c). Furthermore, there were regions of small, disorganised groups of bacteria on the surface, presumably the remains of the inner biofilm (Figure S8d).

### 2.7 Overall Description of a *M. smegmatis* Digitate Colony Protrusion

**FIGURE 5:**
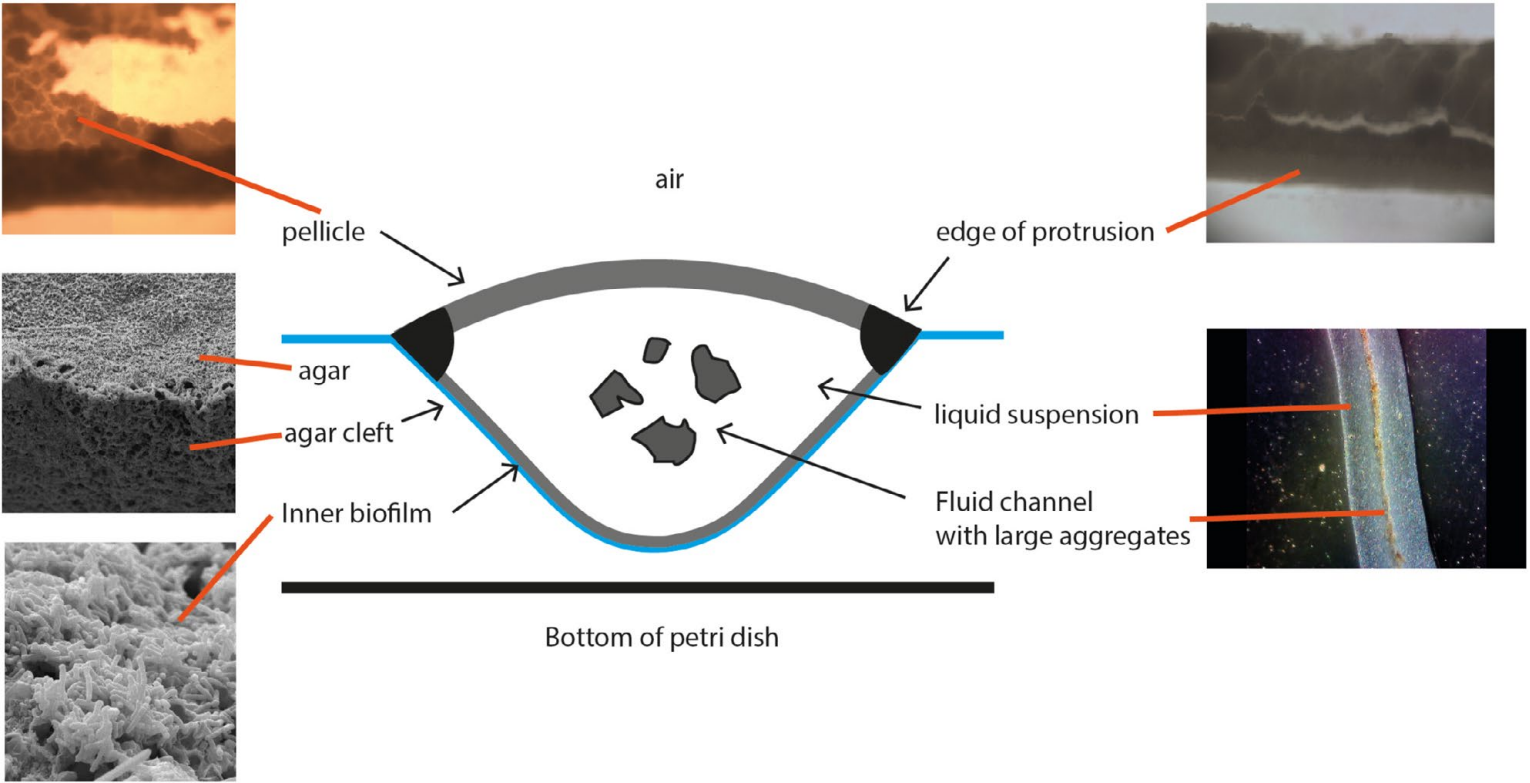
Schematic cross‐sectional representation of a *M. smegmatis* digitate colony protrusion. Two different types of biofilm are present, forming a pellicle on top of the channel fluid and a less robust biofilm at the agar/liquid interface. Within the channel there is a free‐flowing liquid core which contains aggregates of *M. smegmatis*. The protrusion structure sits within a V‐shaped cleft in the agar that extends almost to the bottom of the petri dish.

Based on the observations reported above, we propose a schematic model of a digitate colony protrusion (Figure 5). The liquid core is enclosed by biofilms and physically retained in a V‐shaped groove in the agar. The pellicle is believed to prevent the core from drying out because, in contrast to the biofilm layer, the pellicle can be removed without causing the release of channel fluid. The V‐shaped channel formed in the agar is intriguing as *M. smegmatis* does not have an agarase to digest the agar (Fu and Kim 2010), and so it is likely to be formed by the bacteria pushing aside the agar whilst retaining moisture to expand the channels. The SEM images (Figure S8) support this hypothesis because the agar surface of the channel appears different from the surrounding agar. Additionally, water put onto an agar surface dissipates, even without the action of a surfactant, so fluid must become trapped within the protrusions to prevent it from dissipating (Jain et al. 1991). This proposed structure of a bacterial colony with a liquid core enclosed by biofilm appears to be unique. It remains an open question how *M. smegmatis* acquires and maintains such fluid‐filled channels, especially when the default position for even motile bacteria seems to be to create as physically dense a colony as possible when expanding on agar. The inherent hydrophobicity of the mycobacterial cell envelope may play a part in retaining water in the channels, and the thin biofilm at the interface of the V‐shaped channel likely acts to seal the interface with the agar. The pellicle is also different from other biofilms in that the bacteria formed a liquid‐air biofilm starting from a solid‐air interface (and furthermore, it is derived from pseudofilaments rather than individual bacteria floating in fluid). These novel biofilm structures were not observed for the circular *M. smegmatis* colonies, but common components (such as the GPLs) have been shown to be required for sliding motility in round colonies and formation of conventional *M. smegmatis* biofilms (Recht et al. 2000). It would therefore appear that mycobacterial biofilms and motility are more closely linked than previously expected given the convergence on pellicle formation. This is important as the BCG vaccine is made from harvested pellicle, likely because antigens important for virulence and therefore immunity are expressed in this growth environment.

### 2.8 Fluid Expansion in the Core of the Digitate Colony Protrusions Drives Motility

Having established the overall structure of the digitate colonies, time‐lapse microscopy was used to investigate how these structures formed. The circular colonies moved very much as described previously (Video S3). We did not observe growth of any bacteria over the 12 h period during which the front of the colony was imaged, and the bacteria did not move relative to each other but were pushed out from within the colony. This phenomenon is assumed to arise from unobserved growth at the centre of the colony pushing the bacteria outwards. Martínez et al. (1999) also saw no perceptible growth, but by visually tracking the growth of GFP‐tagged bacteria, they showed the bacteria engaged in 4–6 doublings after 2 days and concluded growth was the basis of the expansion of the circular colonies. With a sufficient thickness of agar, it was also possible to observe the formation of digitate colonies using time‐lapse microscopy. The digitate colonies moved in an unusual fashion (Video S4); whilst the protrusions visually expanded, the only structure that concurrently expanded was the length of the fluid‐filled core. No growth of the aggregates of bacteria or bacterial division in the channel fluid was observed over the 12 h of observation. It is likely that growth was occurring, but it was too slow to directly observe; this would be consistent with previous observations of *M. smegmatis* sliding (Martínez et al. 1999). As the fluid core expanded, it progressively pushed aside groups of the bacteria at the tip. When these bacteria were pushed to the sides, they became condensed and aligned to the central core. We note that there might be some phase‐bright slime at the tip but not at the sides of the protrusions. Unfortunately, with the equipment available to us, it was not possible to track individual bacteria (e.g., by adding 1% GFP‐tagged bacteria) within these 3D structures, but it would be an interesting technical challenge and could generate interesting results (e.g., are there currents within the core suspension, do the aggregates move within the core channel?).

### 2.9 Hydraulic Sliding

In conventional sliding, the bacteria are in direct contact with each other and are pushed away radially from the centre of the colony. It is assumed that the expansive force required to do this is transferred from bacterial growth at the centre of the colony outwards by physical contact between the bacteria, resulting in the bacteria on the edge being pushed outwards. In contrast, in the protrusions of digitate colonies, the bacteria appear to be pushed away from the central fluid core to the sides of the protrusion as the central core advances. The perpendicular sides then apparently become denser (possibly compressed) as the central fluid core moves forward in a linear manner, rather than being pushed outwards in all directions from the centre of the colony. We did not observe growth at the tips, and if this was occurring, we would predict it would be pushing backward or outwards in all directions, which we did not see. The pellicle is not likely to be affecting movement because removing it did not slow down protrusion growth. Within the fluid core, the bacteria are not a uniform compressed mass, so the force cannot be transmitted directly between the bacteria. The biofilms are, however, being pushed away from the liquid core. This suggests that the expansion of the liquid core is the main generator of force. As this is different from conventional sliding but expansive forces are still pushing the bacteria outwards passively, we propose a new type of sliding motility: *hydraulic sliding*. In hydraulic sliding, the bacteria at the tips of the digitate colony protrusions are moved passively by the expansive forces generated in the liquid core, and the edges of the protrusions are retained by biofilms (Video S4). Although water is involved to some degree in facilitating bacterial motility mechanisms, adopting the term hydraulic motility for the behaviour reported here conveys the concept of using contained fluid to push bacteria outwards, similar to a simple hydraulic system. This is distinct from the observations of dendrite motility that were described by Martínez et al. (1999) in a paper that was focused on the circular *M. smegmatis* colonies, in which no liquid channel or enclosing biofilms were reported. Under the conditions used, hydraulic sliding could allow colony expansion that is faster than that allowed by the slow growth rate of mycobacterial species. Notably, the closely related *Streptomyces* species can engage in a specialist explorer form to move faster than their growth rate would permit (Jones et al. 2017).

It remains to be determined why the protrusions formed on Noble agar and not agarose; it could be due to either the overall electrical charge of agarose that interferes with motility or alternatively it could be due to the specific activity of agaropectin. Bacto agar likely has contaminants compared to Noble agar that could inhibit growth, and it is noted that motility and colony expansion are generally sensitive to growth inhibition as there is a feedback loop between growing to expand and accessing more nutrients and then using those nutrients to grow more. Whilst the environmental and physiological signals that trigger digitate colony formation need to be identified before the full biological significance of the behaviour reported here can be determined, it could represent a way for mycobacteria to enclose an environment to: (1) monopolise resources; (2) exclude external predators with biofilms; (3) retain metabolically costly secondary metabolites; and/or (4) create a more beneficial environment for growth.

## 3 Conclusions

Here we report that mycobacteria can engage in a novel form of sliding motility, hydraulic sliding. Expansion of liquid channels contained within protrusions radiating from the centre of digitate colonies pushes the bacteria forwards and outwards, forming stable biofilms around the protrusions. However, while a new type of sliding motility has been observed, the molecular mechanisms that contribute to hydraulic sliding are unknown. Digitate colonies might represent a coordinated response to certain environmental conditions, possibly conceptually similar to the differentiation reported in other bacteria to form specialised sub‐groups and shapes in order to move and form biofilms (Kearns 2010).

## Author Contributions

E.J.G.P., O.C., E.H., and J.G. designed the research. E.J.G.P. and O.C. performed the experiments. E.J.G.P., O.C., E.H., J.G. analysed and interpreted data, and wrote the paper.

## Conflicts of Interest

The authors declare no conflicts of interest.

## Supporting information


**Figure S1:** The formation of digitate colonies is inhibited by higher agar concentrations.
**Figure S2:** Formation of *M. smegmatis* digitate colonies with broad, linear protrusions.
**Figure S3:** Infrared spectra of *M. smegmatis* liquid cultures and digitate colonies.
**Figure S4:** Infrared spectra of *M. smegmatis* grown in liquid culture, digitate colonies and agar.
**Figure S5:** Mass spectrometry principal component analysis (PCA) score plots.
**Figure S6:** SDS‐PAGE analyses of *M. smegmatis* digitate colony extract.
**Figure S7:** Full scale original versions of images used in Figure 2.
**Figure S8:** SEM of the V‐shaped clefts left in the agar after removal of digitate colonies.
**Table S1:** The top metabolite peaks that were higher in the digitate colony samples.
**Text S1:** Experimental procedures and supplementary data.

**Video S1:** Demonstration of fluid mobility of the central core.

**Video S2:** Colony with the pellicle removed.

**Video S3:** Time‐lapse microscopy showing expansion of a round colony.

**Video S4:** Time‐lapse microscopy showing expansion of a digitate colony.

## Data Availability

The data that support the findings of this study are available from the corresponding author upon reasonable request.
