# Supplementary material for: Mycobacterium smegmatis Expands Across Surfaces by Hydraulic Sliding
Source: Environ Microbiol Rep. 2025 Oct 28;17(6):e70214. doi: 10.1111/1758-2229.70214 (PMC12568390; doi:10.1111/1758-2229.70214)
Supplement: Supplementary file 1 — Figure S1: The formation of digitate colonies is inhibited by higher agar concentrations. Figure S2: Formation of M. smegmatis digitate colonies with broad, linear protrusions. Figure S3: Infrared spectra of M. smegmatis liquid cultures and digitate colonies. Figure S4: Infrared spectra of M. smegmatis grown in liquid culture, digitate colonies and agar. Figure S5: Mass spectrometry principal component analysis (PCA) score plots. Figure S6: SDS‐PAGE analyses of M. smegmatis digitate colony extract. Figure S7: Full scale original versions of images used in Figure 2. Figure S8: SEM of the V‐shaped clefts left in the agar after removal of digitate colonies. Table S1: The top metabolite peaks that were higher in the digitate colony samples. Text S1: Experimental procedures and supplementary data. [file EMI4-17-e70214-s004.docx]

**Pollitt et al. Supporting Information**

**Experimental Procedures**

*Motility assay protocols*

The mycobacterium motility assay with 7H9 medium and *M. smegmatis* strain mc^2^155 described by Martínez *et al*. (1999) had to be modified to permit reproducible behaviour under our laboratory conditions. This was not unexpected due to the widely recognized sensitivity of bacterial movement to specific environmental conditions (Tremblay and Déziel, 2008; Patrick and Kearns, 2009).

A range of concentrations of agarose (0.2, 0.3 and 0.4%) as the solidifying agent was tested to obtain circular sliding colonies. The following protocol yielded reproducible circular colonies. The medium was prepared using agarose (Sigma, A5093; 0.4%) and 7H9 (Oxoid, 1.175 g) in 225 ml of distilled water. This was autoclaved and cooled to ~55°C. The medium (25 ml aliquots) was then dispensed into 9 cm Petri dishes. The plates were left covered in a laminar flow cabinet overnight (not running) to set and then inoculated with colonies taken directly from an *M. smegmatis* stock plate. The plates were then sealed with Parafilm and incubated at 37°C in a humidity-controlled water jacketed incubator (Thermo Scientific Forma 3120). Colonies were visible and started to expand after 2 days, and growth ceased by day 7. It was important to ensure that vibrations were kept to a minimum to maintain the integrity of the plates over the extended time course of the assay.

For the formation of digitate colonies, it was found that Bacto agar (BD), our available equivalent to the Difco agar used by Martínez *et al*. (1999) did not yield satisfactory results. Therefore, a range of concentrations of Noble agar (Oxoid; 0.2, 0.25 and 0.3%) was tested. Based on preliminary experiments the following protocol was adopted. Noble agar (Oxoid; 0.25%) and 7H9 medium (Oxoid, 1.175 g) was added to distilled water (225 ml) and autoclaved. The sterile medium was dispensed into petri dishes, stored, inoculated and incubated as described above.

*Light microscopy*

*M. smegmatis* colonies were analysed using a range of microscopic techniques. A benchtop Zeiss microscope was used for observation of bacteria within the colony; a dissecting microscope was used for observing overall colony morphology; and a Nikon Eclipse Ti2 wide field microscope was used for time-lapse recordings of the movement of the edge of the colony. To facilitate time-lapse microscopy, 2 ml of medium in glass windowed mini 35 mm Petri dishes (Nunc) permitted digitate and circular colony formation, whilst allowing focussing on the top surface of the agar from below using a Nikon Eclipse Ti2 wide field microscope with a 10x objective. The focal drift compensation mechanism was used to keep the colony in focus during the overnight time-lapse sequence.

In some experiments the colonies were physically manipulated using a 200 µl pipette tip. Where we compressed the samples under the microscope, we first excised a section of the colony by sliding a glass slide underneath and cutting it out of the agar plate with a coverslip. Then, whilst viewing it with the microscope (so we could visually track the structures), we put a coverslip over the top and compressed the sample keeping the structures of interest in view. All images are representative of three different independent replicates and microscopy images are shown with the appropriate scale bars.

*Scanning electron microscopy (SEM)*

A section of the colony was removed by sliding a glass slide underneath and cutting it out of the agar plate with a coverslip. Sections (2x2 cm) were fixed overnight in 2.5% glutaldehyde/0.1 M sodium cacodylate buffer. The following day they were washed in buffer, post fixed in 2% aqueous osmium tetroxide and dehydrated in a graded ethanol series and dried in 50% hexamethyldisilazane (HEX) in ethanol. Final drying was in 100% HEX. After removal from the final HEX wash, sections were left to dry overnight, in a fume hood. Samples were mounted onto a pin-stub using a Leit-C sticky tab, gold coated using an Edwards S150B sputter coater and examined in a Tescan Vega3 LMU scanning electron microscope.

*Extraction and processing of digitate colony fluid cores*

The fluid cores of the protrusions from multiple colonies (at least 10 combined) grown for 3 days were extracted using a 1000 µl pipette tip, resulting in 20 ml of fluid being available for each analysis. The fluid was freeze dried using a ScanVac Cool Safe 55-4 Pro 3800 for 2 days. For comparison, medium plus agar (20 ml), liquid 7H9 media (20 ml), and liquid *M. smegmatis* culture grown for 3 days (20 ml) were also processed. The samples were analysed by Infrared (IR) and mass spectroscopies, as well as separating any proteins by SDS-polyacrylamide gel electrophoresis (SDS-PAGE).

*IR spectroscopy*

The freeze-dried powders were directly analysed using a PerkinElmer Spectrum One Spectrometer with a diamond ATR sampling accessory (eight scans per sample with a resolution of 4 cm^-1^). The powder was removed after measurement using a strong detergent and the plate was cleaned with distilled water before loading the next sample (as the samples tended to strongly adhere to the surfaces and diamond of the spectrometer).

*Mass spectrometry*

The solid processed samples were dissolved in a 1:1 mixture of ultrapure water and ultrapure methanol and analysed by directly injecting into a Waters G2 Synapt Mass Spectrometer in both positive and negative ion mode. Peak lists of m/z versus ion counts were generated and manipulated to remove background noise and the data was then binned to 0.2 amu according to the procedure by Overy *et al*. (Overy *et al.*, 2005). Principal component analysis plots were generated from these peak lists. The top peaks that were higher in the colony sample and not the control sample were provisionally matched with corresponding masses in the *Escherichia coli* metabolome (ecocyc.org) and mycobacterial component mass matrix (Zampieri *et al*., 2018). Three technical replicates were obtained by analysing the sample three times.

*Protein analysis*

Freeze dried samples (see above) were re-suspended in water (100 μl) and mixed with SDS-PAGE loading buffer (1:1), containing 10% (v/v) 2-mercaptoethanol. After boiling for 15 min, the samples were loaded onto a 15% SDS-PAGE gel (with a 4% stacking gel). Polypeptides were separated by electrophoresis at 150 V for 70 min. Polypeptides were stained with Generon Quick Coomassie and imaged on a Gbox Chemi-XX9 imager (Syngene).

**Supplementary Data**

*Chemical analysis of the channel fluid*

We assessed the feasibility of analysing the core channel fluid using IR and mass spectroscopies to advance our understanding of the chemical basis of the biofilm and channel formation. This revealed there were substantial chemical differences in the digitate colonies compared to liquid cultures, and very likely different compounds were also present (Fig. S3, 4). The IR spectroscopy with its different peaks shows different bond arrangements are present. However, no positive identifications of digitate colony channel fluid components could be made as the spectroscopic peaks overlap in the 200 - 400 cm^-1^ region where many different bond types are present (Fig. S3, 4). Mass spectroscopy PCA score plots showed that the digitate colony samples were fundamentally different from the liquid culture samples despite the same nutritional source (Fig. S5). Putative matches were initially identified against the *E. coli* metabolite database ([ecocyc.org](http://ecocyc.org)) for the most abundant digitate colony peaks (Table S1) (Keseler *et al.*, 2017). Remaining unknown peaks were compared to mycobacteria reference mass lists (Zampieri *et al.*, 2018). Some of these putatively identified compounds are directly associated with the mycobacterial cell envelope such as p-HBAD (associated with the phenolic glycolipids, a major component of the cell envelope) and Biotinyl-5-AMP (biotinylation being required for the Mycobacterial cell wall). The SDS-PAGE analysis did not reveal major differences in protein content between the *M. smegmatis* liquid culture and the digitate colony extract with a prominent species of ~150-200 kDa being detected in both samples (Fig. S4). Nevertheless, we have shown that the digitate colony channel fluid can be readily extracted and chemically characterised, which should enable further studies to identify the major components of the channel fluid.

**Supplementary video files**

**Video S1.** Demonstration of fluid mobility of the central core. Rocking the plate backwards and forwards shows how readily the fluid moves within the central core (as can be seen by the aggregates moving within it).

**Video S2.** Colony with the pellicle removed. Rocking of the protrusion with the pellicle removed shows there is a layer at the bottom of the protrusion. The bacterial suspension can be seen moving backwards and forwards as it is rocked over the bottom layer of bacteria.

**Video S3.** Time-lapse microscopy showing expansion of a round colony. Video showing time-lapse microscopy of the expansion of a round colony over the course of 4 h 40 min. The bacteria are pushed forward evenly from behind by the main mass of the colony.

**Video S4.** Time-lapse microscopy showing expansion of a digitate colony. Video showing time-lapse microscopy of the expansion of a digitate colony protrusion over the course of 8 h.

**Supplementary Figures**

| **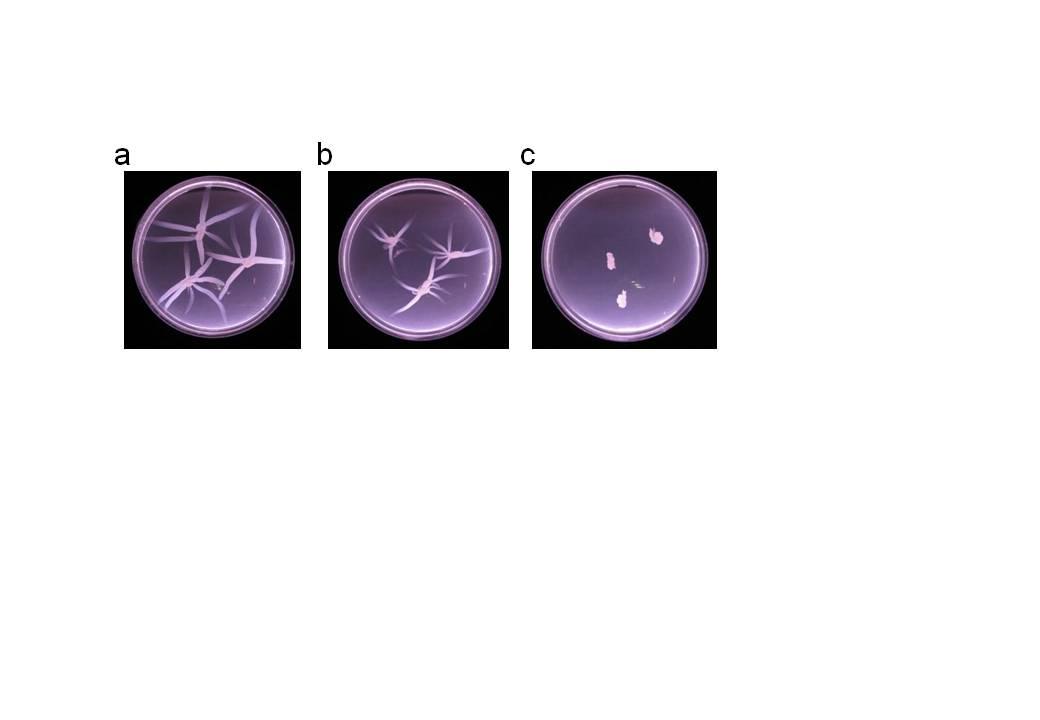** |
| --- |
| **Fig. S1.** The formation of digitate colonies is inhibited by higher agar concentrations. Images of *M. smegmatis* colonies grown on 7H9 medium solidified with (a) 0.2% Noble agar; (b) 0.25% Noble agar; and (c) 0.3% Noble agar. All images were obtained 3 days post-inoculation. The media were prepared as described in *Supplementary Experimental Procedures*. |

| **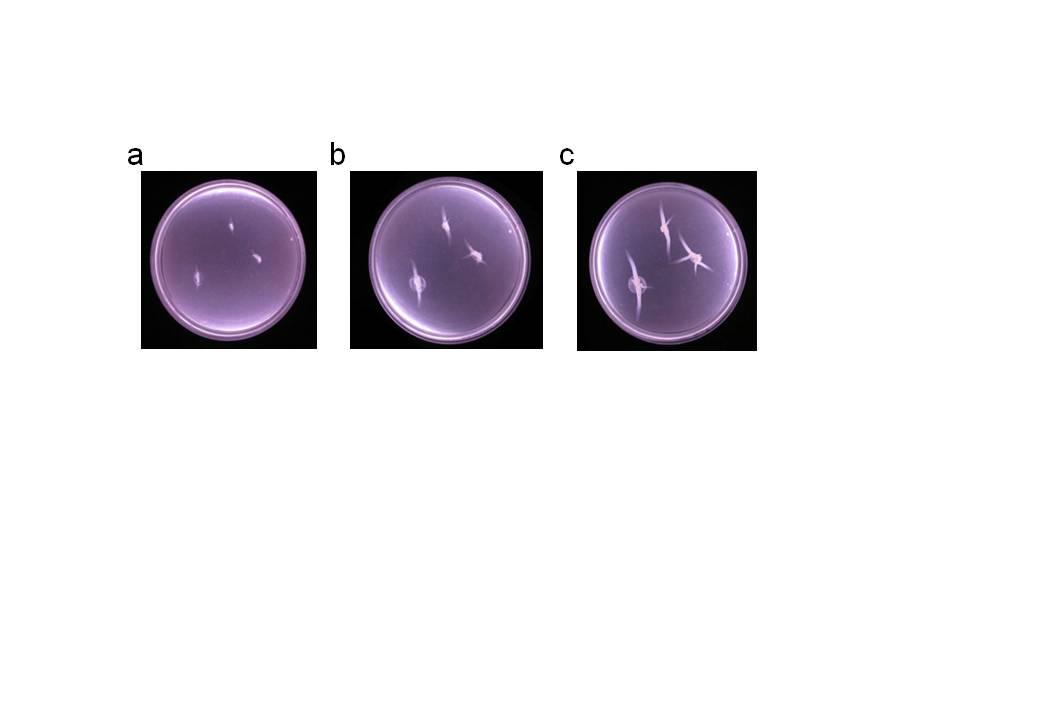** |
| --- |
| **Fig. S2.** Formation of *M. smegmatis* digitate colonies with broad, linear protrusions. The representative agar plate shown was inoculated at three points with *M. smegmatis* and incubated at 37°C. The plate was imaged: (a) 24 h; (b) 48 h; and (c) 72 h, post-inoculation. The medium was: Noble agar (Oxoid; 0.25%) and 7H9 medium (Oxoid, 1.175 g) in distilled water (225 ml) prepared as described in *Supplementary Experimental Procedures*. |

| **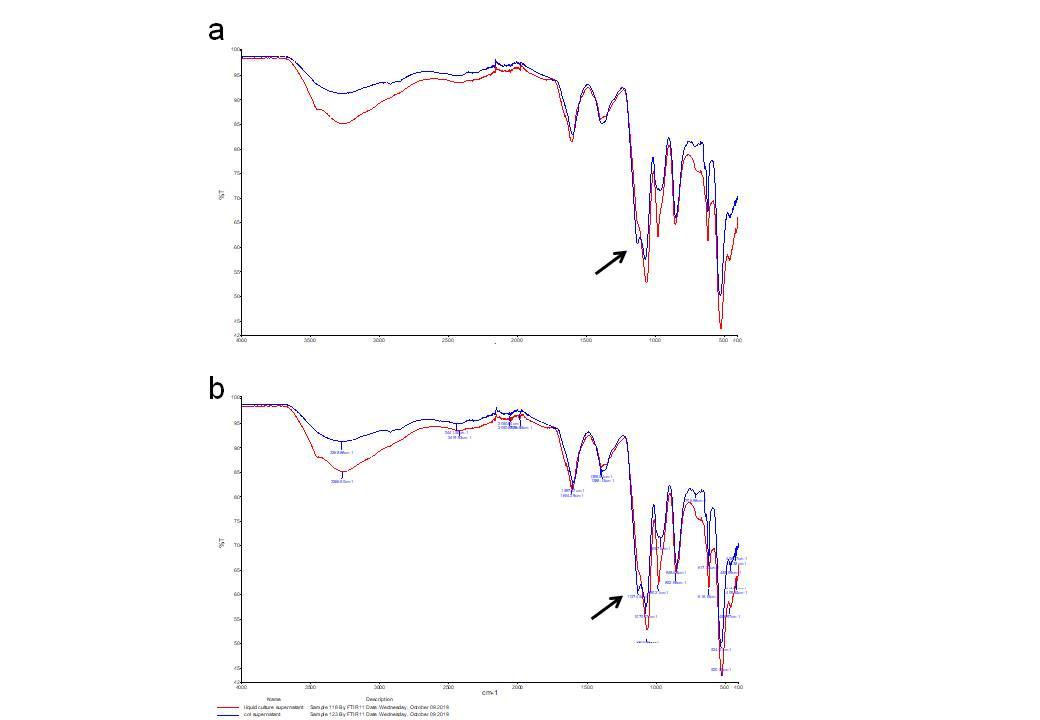** |
| --- |
| **Fig. S3.** Infrared spectroscopy spectra of *M. smegmatis* liquid cultures and digitate colonies. (a) Note that overall the spectra (liquid culture, red trace; digitate colony, blur trace) are similar, but below 2000 cm^-1^ there are dissimilar peaks (arrowed) reflecting the presence of different compounds. (b) Annotated (wave number) versions of each trace. For experimental details see *Supplementary Experimental Procedures*. |

| **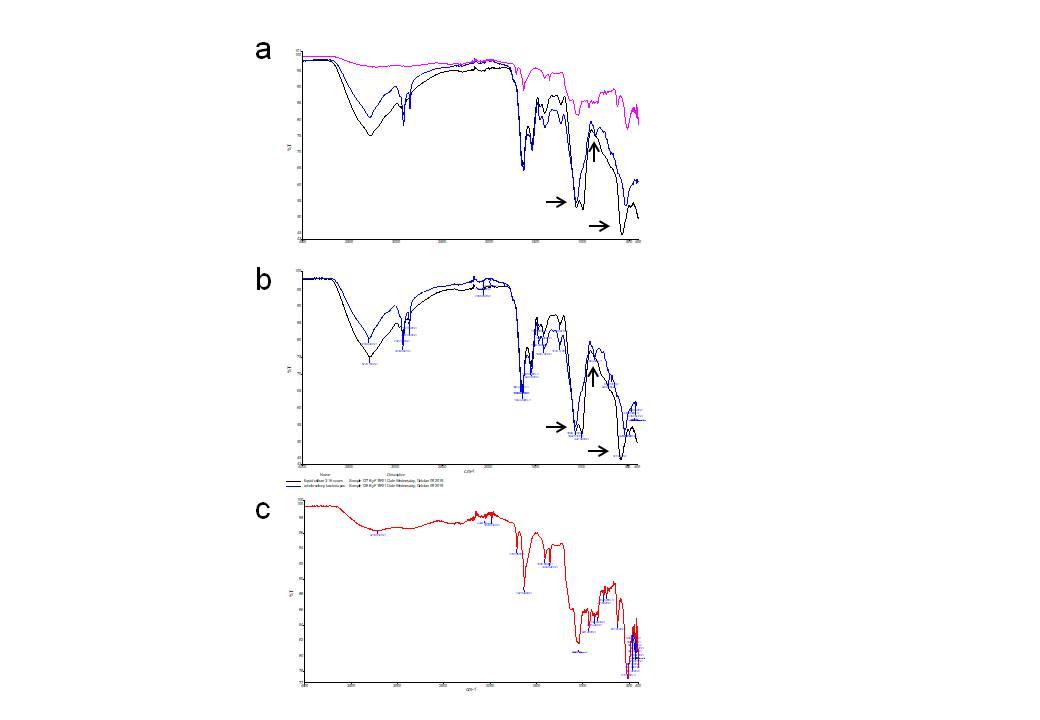** |
| --- |
| **Fig. S4.** Infrared spectra of *M. smegmatis* grown in liquid culture, digitate colonies and agar. Spectra shown are those of the liquid culture bacterial pellet (black trace), digitate colony bacterial pellet (blue trace) and agar (purple trace). There are substantial differences below 2000 cm^-1^ likely indicating different bond arrangements and therefore different compounds (arrowed). (b and c) Annotated (wave number) versions of each trace. For experimental details see *Supplementary Experimental Procedures*. |

| **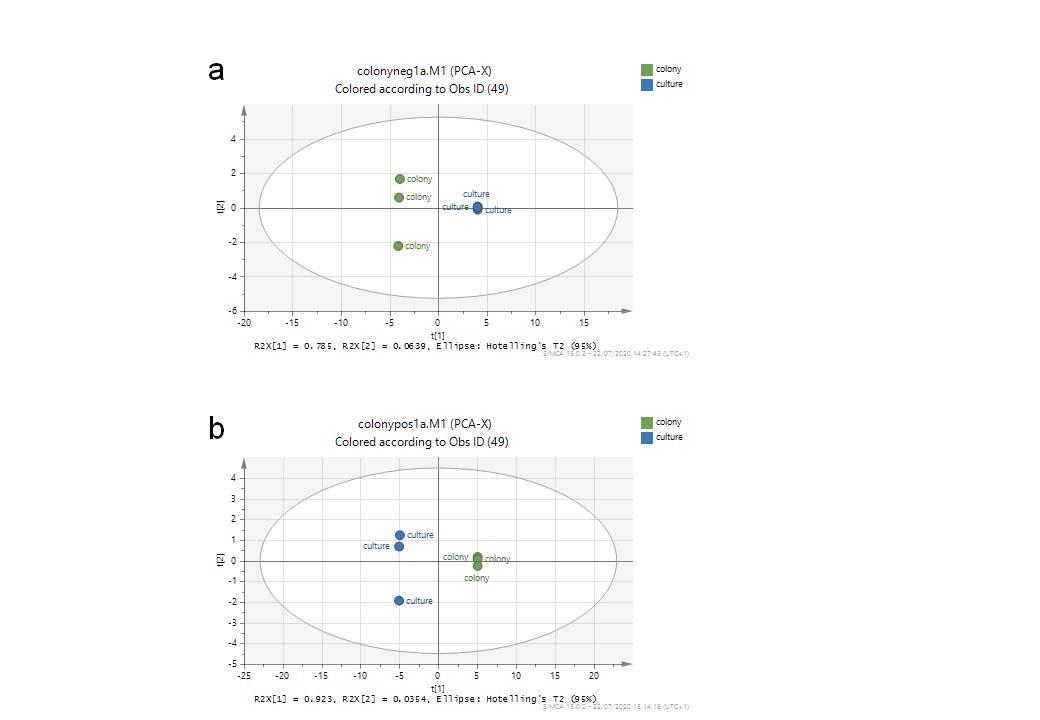** |
| --- |
| **Fig. S5**. Mass spectrometry principal component analysis (PCA) score plots. Three technical replicates were used for the analysis. (a) Positive ion mode. (b) Negative ion mode. The green circles indicate digitate colony fluid samples (colony) and the blue circles liquid culture medium samples. For experimental details see *Supplementary Experimental Procedures*. |

| 1  2 3  4  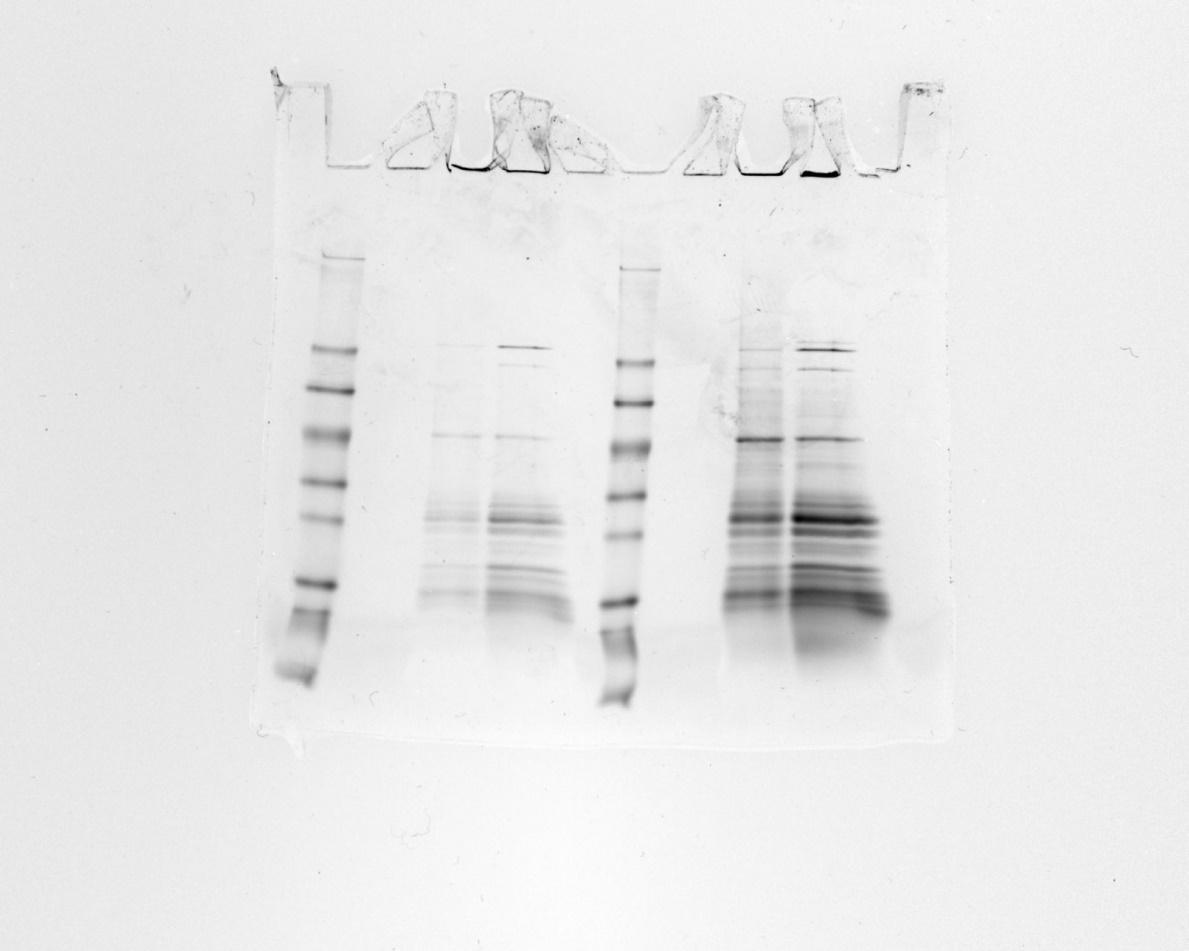 |
| --- |
| **Fig. S6.** SDS-PAGE analysis of *M. smegmatis* digitate colony extract. Lanes (1) NEB ladder unstained protein standard, Broad Range; (3) 7H9 medium control; (4) *M. smegmatis* liquid culture; (5) *M. smegmatis* digitate colony extract. For experimental details see *Supplementary Experimental Procedures*. |

| **a**  **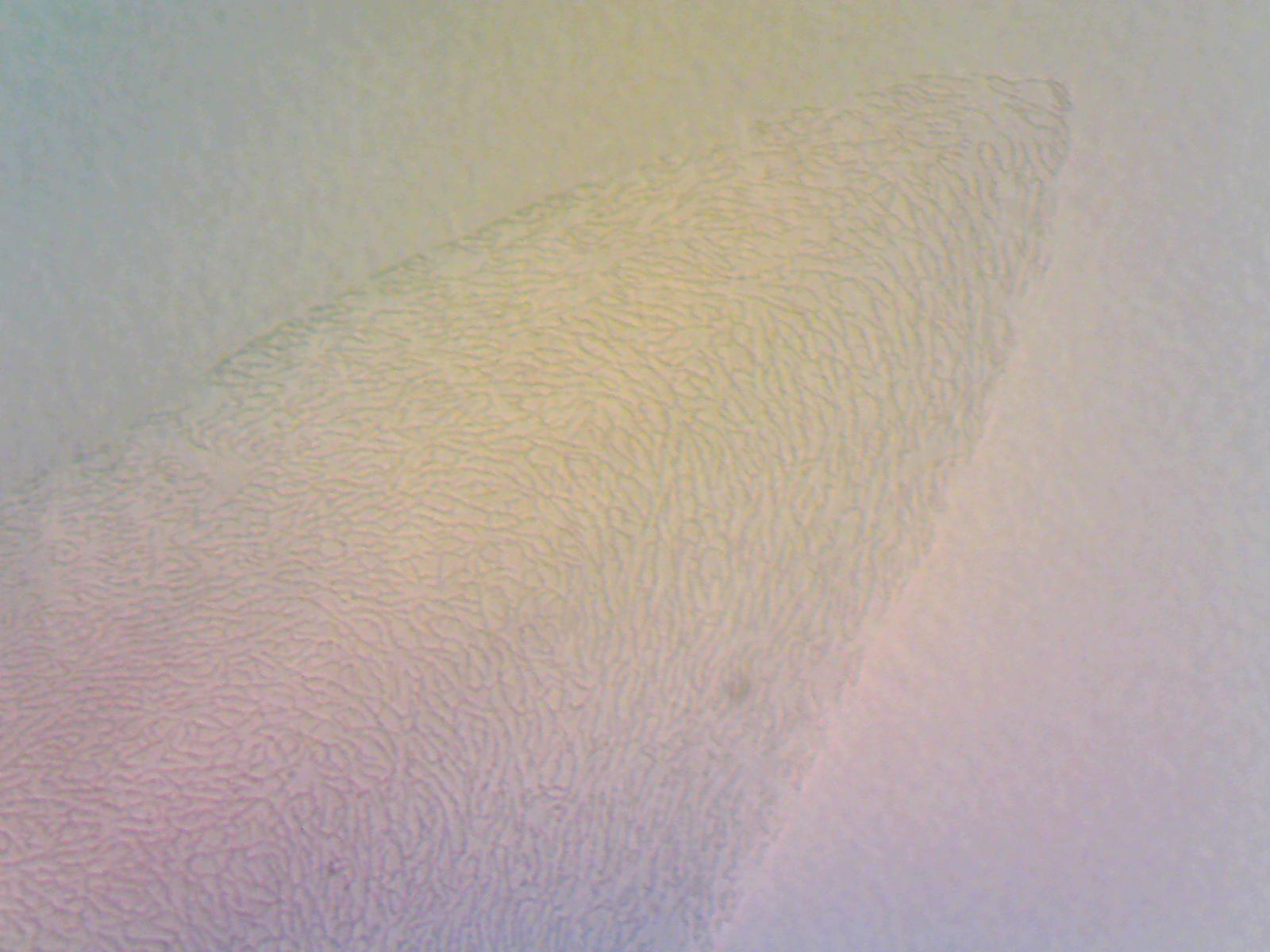**  **b**  **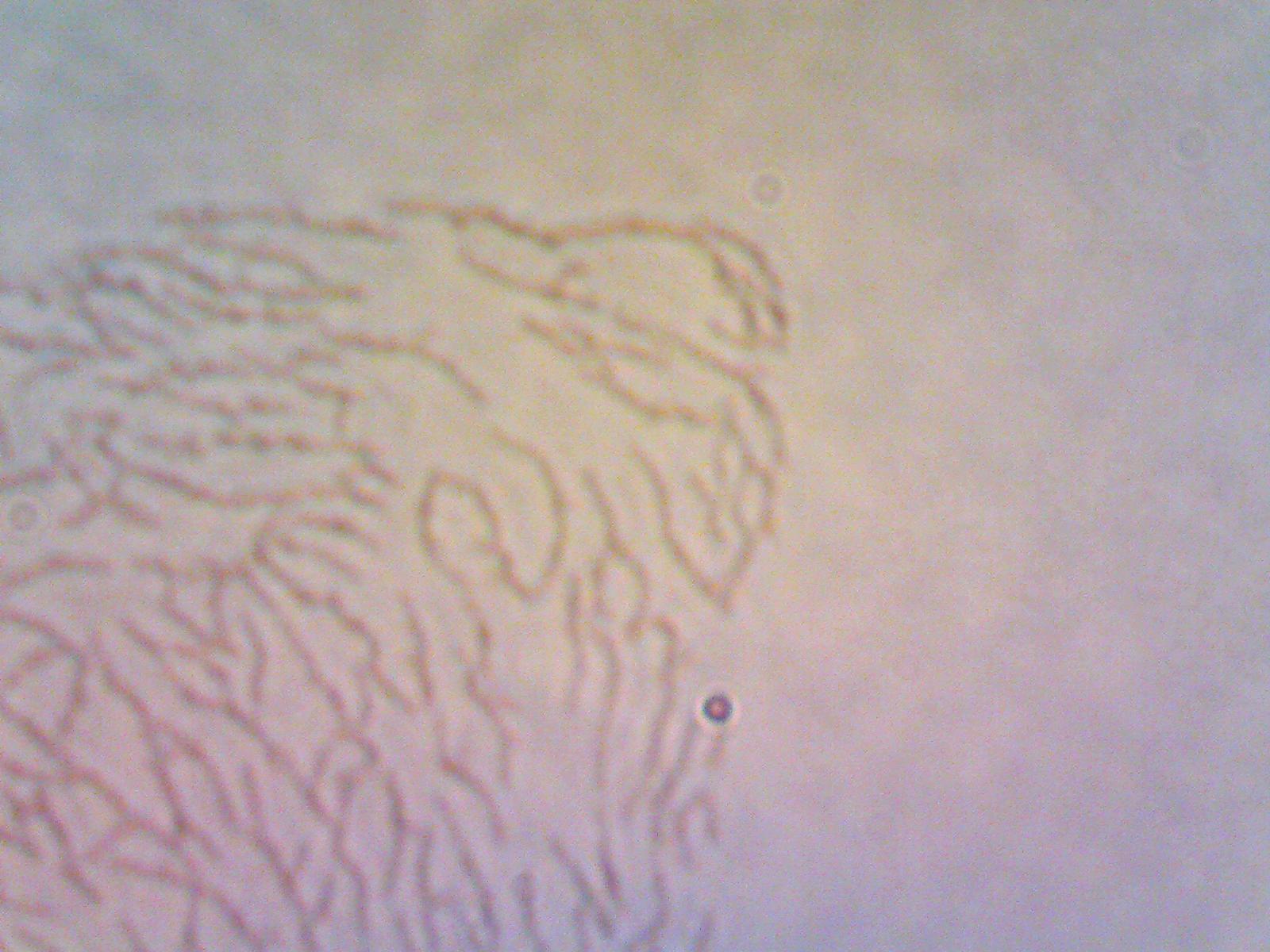**  **c**  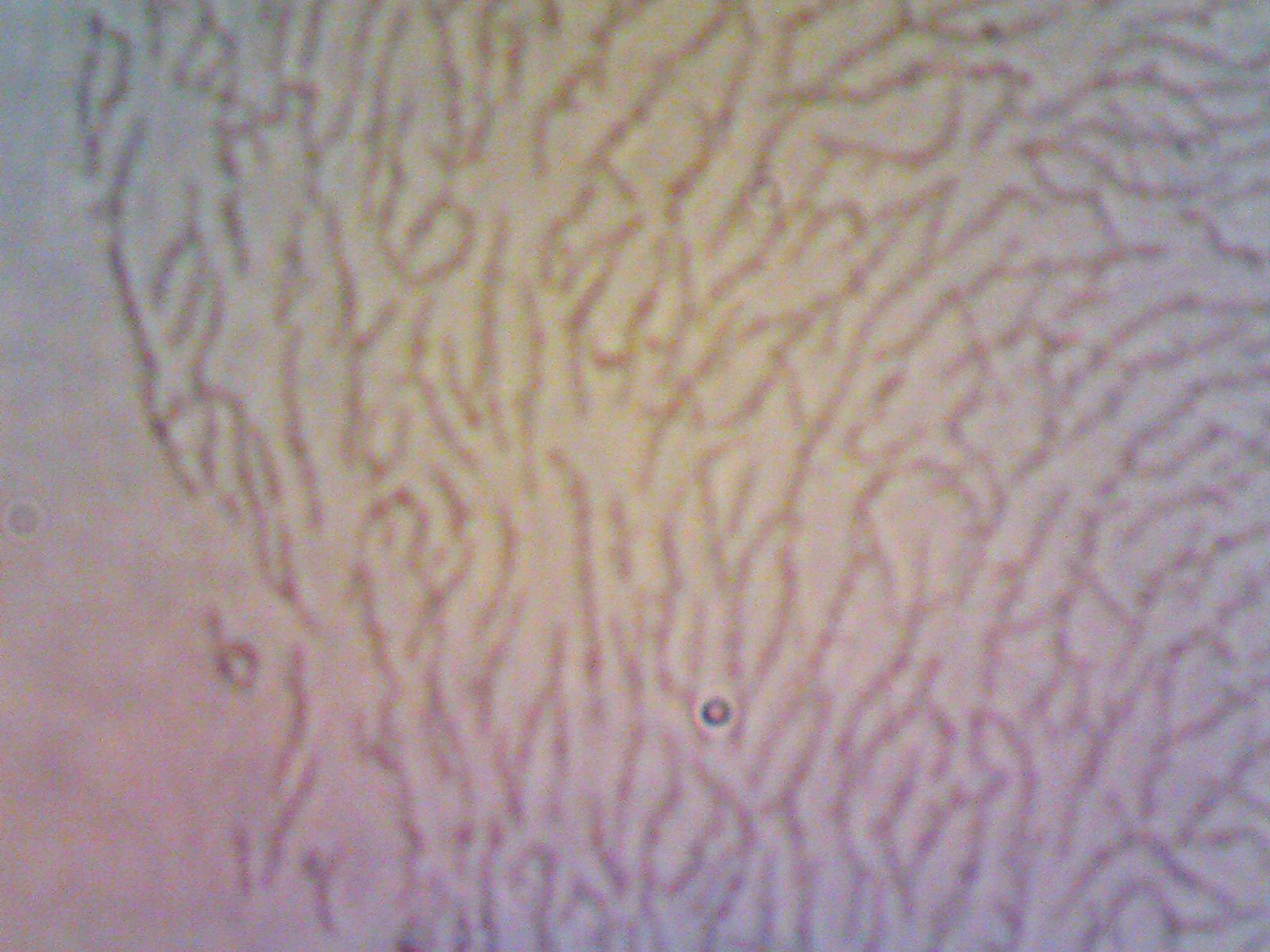  **d**  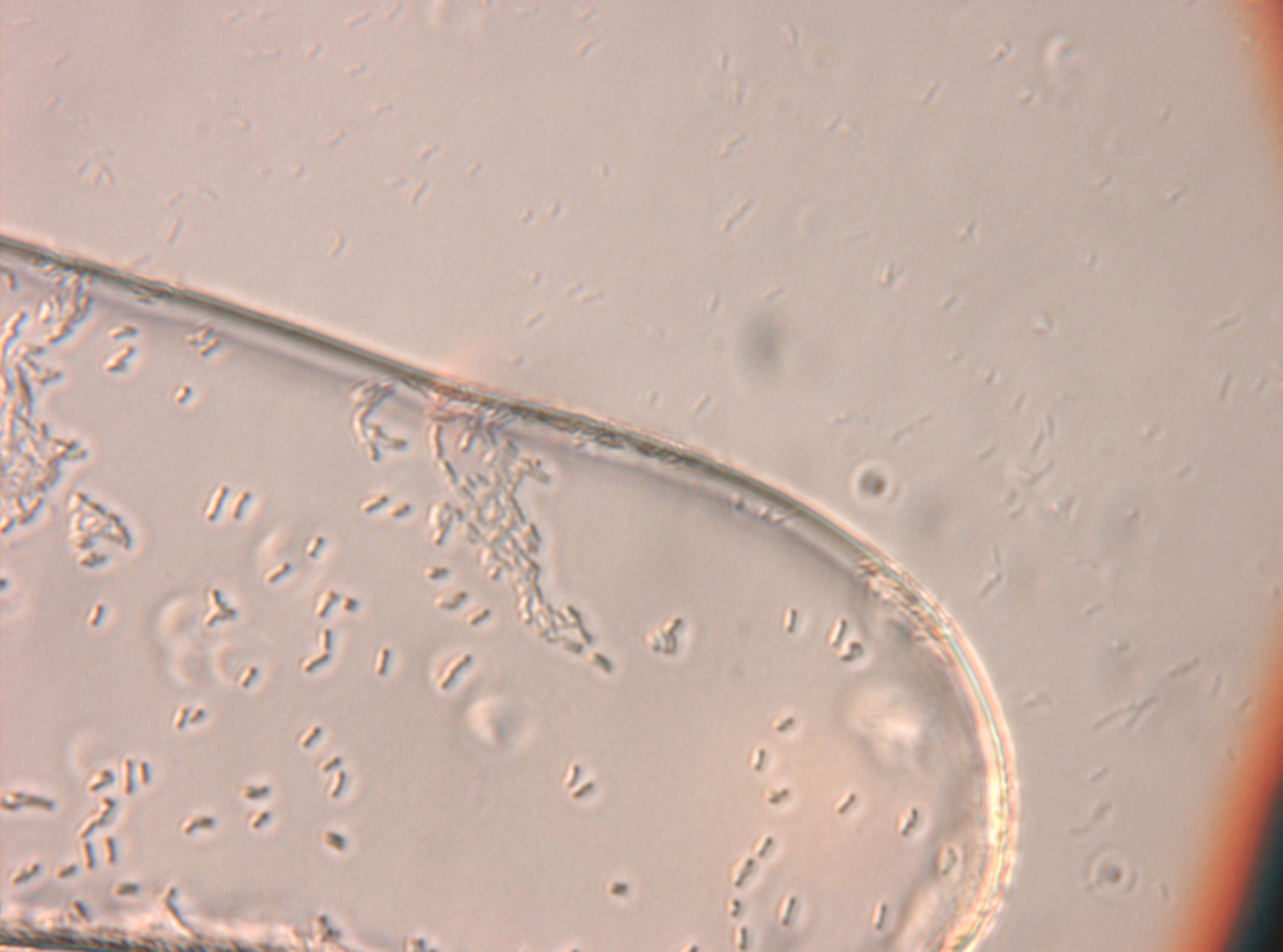  **e**  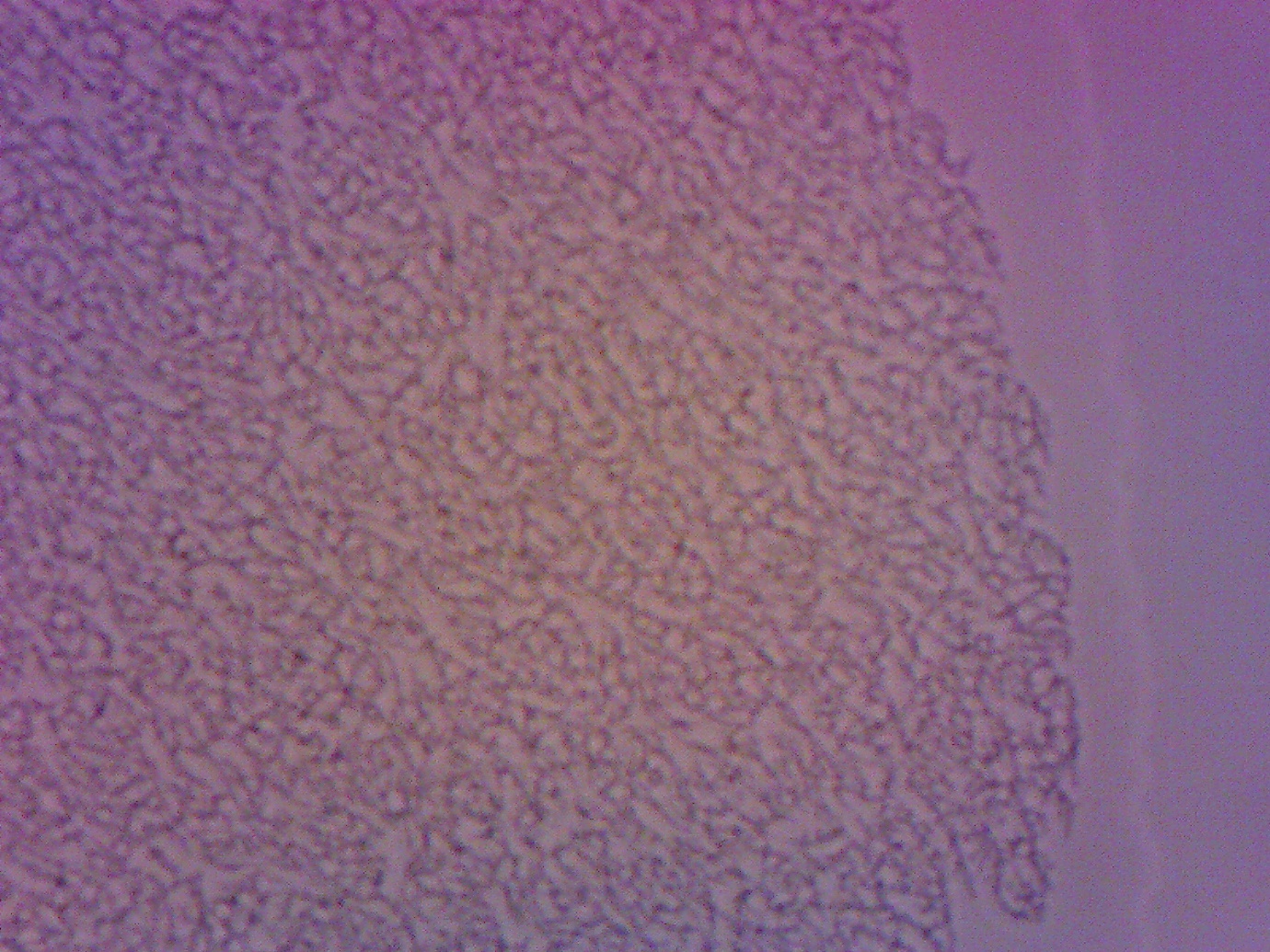  **f**  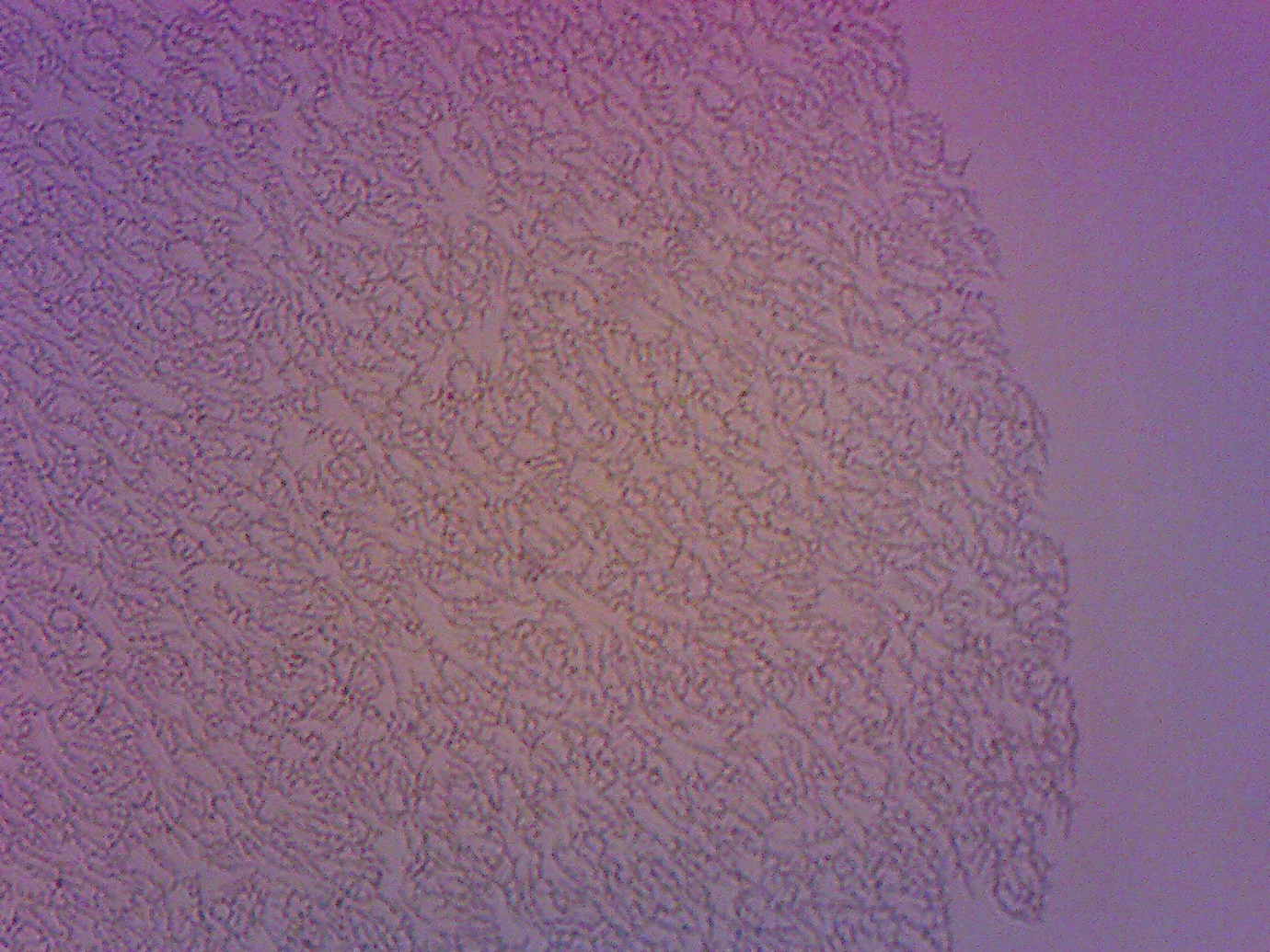 |
| --- |
| **Fig. S7.** Full scale original versions of images used in Fig 2. Light microscopy images of *M. smegmatis* sliding colonies. (a) The tip of a digitate colony observed using a phase contrast microscope. The bacteria are arranged as branching pseudofilaments that broadly align with the curve of the protrusion tip. Scale Bar: 100 µm. (b) A higher magnification view of the pseudofilaments at the tip is shown. Scale Bar: 25 µm. (c) A higher magnification view of the side of the colony showing how the pseudofilaments align with the edge of the colony. Scale Bar: 25 µm. (d) Image of *M. smegmatis* cells grown in 7H9 liquid culture on a glass slide showing that the cells do not branch. Scale Bar: 50 µm. (e) The edge of a circular *M. smegmatis* colony showing the pseudofilaments are unaligned and the fluid surrounding the colony. Scale Bar: 50 µm. (f) A focused image used to obtain a digitally higher magnification image of the unaligned pseudofilaments – a physically higher magnification image was not achievable due to the amount of fluid surrounding the circular colonies. Scale Bar: 50 µm. |

| 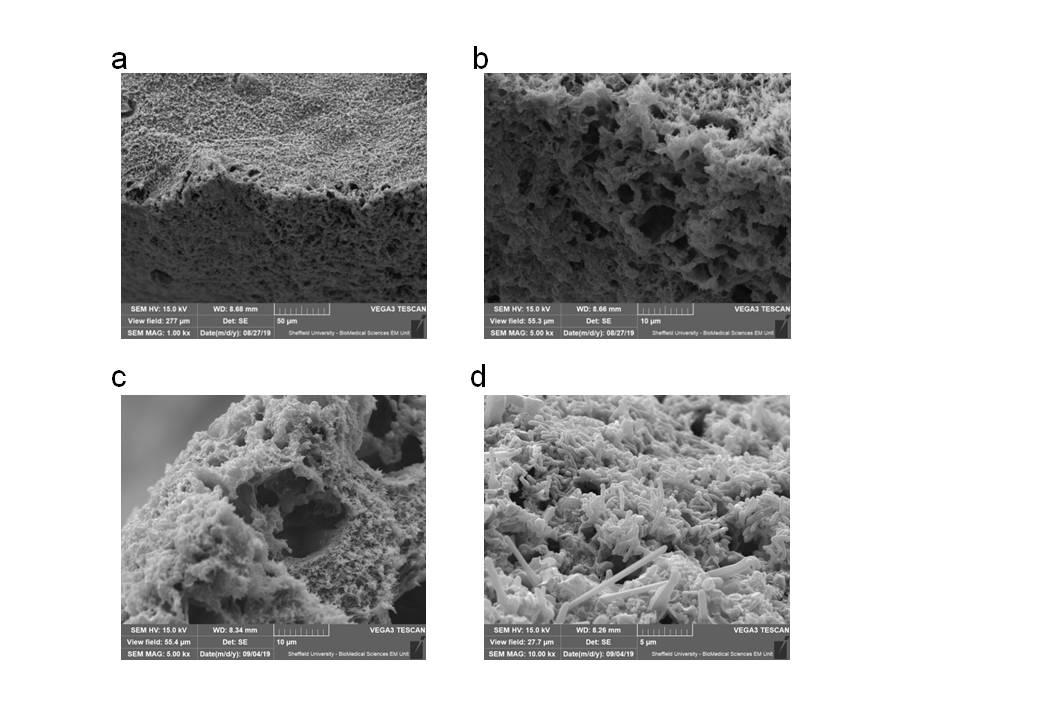 |
| --- |
| **Fig. S8.** SEM of the V-shaped clefts left in the agar after removal of digitate colonies. The SEM fixation process removed the pellicle and contents of the protrusions permitting observation of the inner surface. (a) The top of the image shows the agar surface, whilst the bottom shows the inside of the protrusion which has a different consistency. (b) The same as (a) but at higher magnification. (c) The edge taken from the opposite angle. The voids are presumed to be where water is being lost. (d) A patch of bacteria on the inside of the protrusion. For experimental details see *Supplementary Experimental Procedures*. |

**Supplementary Table**

| **Table. S1.** The top metabolite peaks that were higher in the digitate colony samples. | |
| --- | --- |
| **Negative Mode - Bin** | **Possible ID's** |
| 191 | citrate/isocitrate/quinate* |
| 333 | 2-(alpha-D-galactosyl)-sn-glycerol 3-phosphate |
| 282.8 | Guanosine* |
| 221 | cystathionine/flavone* |
| 212 | L-aspartyl-4-phosphate* |
| 712.6 | -2-Octaprenyl-3-methyl-5-hydroxy-6-methoxy-1.4-benzoquinone ** |
| 802.8 | - Phenolpentacosanoyl AMP ** |
| 355 | 5-amino-6-(5'-phosphoribitylamino)uracil* |
| 111 | Uracil* |
| **Positive Mode - Bin** | **Possible ID's** |
| 288.8 | - |
| 430.8 | -N6-Hydroxy-L-lysine coupled to octadecanoyl group# |
| 572.8 | - Biotinyl-5-AMP # |
| 171 | glyceraldehyde-3-phosphate/glycerone phosphate/ornithine/asparagine* |
| 732.6 | - Phosphatidylethanolamine # |
| 448.8 | -Geranylgeranyl diphosphate (GGPP) # |
| 784.8 | - Flavin-adenine dinucleotide-oxidized ** |
| 165 | fucose and hexose deoxy sugar isomers/S-methylmethionine* |
| 590.8 | - Glycosylated p-HBAD I # |

Samples marked * have been putatively identified as matching a corresponding mass in the *E. coli* metabolome (ecocyc.org). After this unknown samples marked ** and # were further putatively matched against a Mycobacteria reference mass list (Zampieri *et al.*, 2018). # marked samples can be associated with the mycobacterial cell envelope. Samples marked – no match was found.

**References**

Keseler, I.M., Mackie, A., Santos-Zavaleta, A., Billington, R., Bonavides-Martínez, C., Caspi, R., , Fulcher C., Gama-Castro S., Kothari A., Krummenacker M., Latendresse M., Muñiz-Rascado L., Ong Q., Paley S., Peralta-Gil M., Subhraveti P., Velázquez-Ramírez D.A., Weaver D., Collado-Vides J., Paulsen I., Karp P.D.*et al.* (2017) The EcoCyc Database: Reflecting New Knowledge About *Escherichia coli* K-12. Nucleic Acids Research 45: D543–D550.

Martínez A. Torello S. Kolter R. 1999 Sliding Motility in Mycobacteria. Journal of Bacteriology 181 7331-7338

Overy, S.A., Walker, H.J., Malone, S., Howard, T.P., Baxter, C.J., Sweetlove, L.J., et al. (2005) Application of Metabolite Profiling to the Identification of Traits in a Population of Tomato Introgression Lines. Journal of Experimental Botany 56: 287–296.

Patrick and Kearns, 2009). Patrick, J.E. and Kearns, D.B. (2009) Laboratory Strains of *Bacillus subtilis* do Not Exhibit Swarming Motility. Journal of Bacteriology 191: 7129–7133.

Tremblay J. Déziel E. 2008 Improving the Reproducibility of *Pseudomonas Aeruginosa* Swarming Motility Assays Journal of Basic Microbiology 48 509 515 (Zampieri *et al.*, 2018).

Zampieri, M., Szappanos, B., Buchieri, M.V., Trauner, A., Piazza, I., Picotti, P., et al. (2018) High-Throughput Metabolomic Analysis Predicts Mode of Action of Uncharacterized Antimicrobial Compounds. Science Translational Medicine 10: (429):eaal3973
